# PRIORITY IR: Protocol for implementation research on single-dose postpartum IV iron to treat iron-deficiency anemia among women in India and Pakistan

**DOI:** 10.12688/gatesopenres.16352.1

**Published:** 2025-07-15

**Authors:** Valerie L. Flax, Narjis Rizvi, Umesh Charantimath, Saleem Jessani, Avinash Kavi, Sarah Saleem, Manjunath Somannavar, Shivaprasad S. Goudar, Anika Hannan, Elizabeth M. McClure, Simal Thind, Rosemary Frasso, Richard Derman

**Affiliations:** 1 RTI International, 3040 E Cornwallis Road, Research Triangle Park, North Carolina, 27709, USA; 2 The Aga Khan University, National Stadium Road, Karachi, Karachi City, Sindh, Pakistan; 3 Jawaharlal Nehru Medical College, KLE Academy of Higher Education and Research, Nehru Nagar, Belagavi, Karnataka, 590010, India; 4 Thomas Jefferson University, 901 Walnut Street, Philadelphia, Pennsylvania, 19107, USA; 5 Sidney Kimmel Medical College, Thomas Jefferson University, 1025 Walnut Street, Philadelphia, Pennsylvania, 19107, USA; 6 Thomas Jefferson University, 1020 Walnut Street, Philadelphia, Pennsylvania, 19107, USA

**Keywords:** anemia, iron deficiency, mothers, intravenous iron, postpartum, implementation research, India, Pakistan, low- and middle-income countries

## Abstract

**Background:**

Anemia among women of reproductive age has remained highly prevalent globally. Intravenous (IV) iron is well tolerated and proven effective for treating postpartum iron deficiency anemia in high-income countries, but evidence from LMICs, where oral iron is standard treatment, is limited. The PRIORITY trial will test the effectiveness of IV iron compared to oral iron for postpartum women with moderate anemia in eight LMIC sites. An implementation research (IR) study will be conducted alongside the PRIORITY trial in India and Pakistan to gather information on the intervention characteristics and the implementation process, and to assess feasibility, acceptability, fidelity, and cost of implementation for providing IV iron to postpartum women with moderate iron deficiency anemia.

**Methods:**

The PRIORITY IR study will use a mixed methods convergent parallel design guided by two frameworks: the Consolidated Framework for Implementation Research and Proctor’s implementation outcomes. The IR study will be conducted in the Belagavi, India and Karachi, Pakistan PRIORITY trial sites. Participants will include postpartum women in the IV iron intervention arm of the trial, family members, health workers administering IV iron, hospital administrators, postpartum women who refuse to be part of the trial (Pakistan only), and postpartum women in the oral iron arm of the trial (India only). Data collection methods will include surveys, in-depth interviews, a supervision checklist, and a cost assessment. Survey and supervision checklist data will be analyzed descriptively. Interview data will be analyzed using a directed content analysis approach.

**Discussion:**

The PRIORITY IR study will contribute important information about implementation processes and strategies and feasibility, acceptability, fidelity, and costs for postpartum IV iron implementation. Results of the study can provide guidance for implementing effective anemia treatment in LMIC contexts with a high anemia burden.

**Registration:**

NCT05590260 (21/10/2022), CTRI/2022/10/046632 (19/10/2022), CTRI/2023/05/053302 (31/05/2023).

## Introduction

Anemia continues to affect more than 40% of women of reproductive age globally, ^1^ and iron deficiency is the primary etiology in low- and middle-income countries (LMICs). Anemia during pregnancy is a risk factor for maternal mortality and poor birth outcomes, with the highest burden in LMICs. ^2, 3^ To address this major public health challenge, the World Health Assembly has called for a 50% reduction in the prevalence of anemia in women of reproductive age. ^4^ Anemia prevalence in these settings has remained relatively stagnant or even increased despite the availability of oral iron treatment, ^1, 5^ indicating the need for a change in approach. The standard of care to treat confirmed cases of iron deficiency anemia during pregnancy and postpartum in most LMICs is daily iron and folic acid (IFA) tablets, the use of which has persistent challenges in distribution and adherence. ^6– 8^ In high-income countries, intravenous (IV) iron is widely available in a single dose infusion and increasingly used to treat moderate anemia during pregnancy and postpartum. In LMICs, the use of IV iron during pregnancy and postpartum presents a novel, innovative opportunity to rapidly treat moderate iron deficiency anemia. This approach could overcome distribution and adherence challenges associated with IFA and could potentially improve maternal and newborn outcomes.

Although there is evidence that different formulations of IV iron are effective at improving hematologic outcomes postpartum, the sample sizes of many of these studies are small and other important maternal outcomes associated with an anemic state, such as depression and quality of life, have not been adequately studied. ^9– 11^ The Prevention of I ron Def iciency Anemia Pos t-Deliver y (PRIORITY) trial is a large 2-arm randomized controlled trial (N=4,800) designed to evaluate the effectiveness of a single dose of ferric carboxymaltose (FCM) IV iron versus oral iron in reducing anemia among postpartum women with moderate anemia (Hb 7.0 to 9.9 g/dL). The trial is being conducted at eight sites across seven countries (Bangladesh, India [2 sites], Pakistan, Democratic Republic of Congo, Kenya, Zambia, and Guatemala). The primary outcome is maternal non-anemic state (Hb ≥11 g/dL) at 6 week post-delivery and includes a range of secondary outcomes, such as post-discharge blood transfusion, postpartum hemorrhage requiring transfusion or major surgery, maternal and infant hospitalization, postpartum depression, maternal fatigue, mother-infant bonding, quality of life, and exclusive breastfeeding. Details of the rationale and study design for the PRIORITY trial can be found in Derman et al. ^12^

If postpartum IV iron is proven effective through PRIORITY and other trials, it will become another important tool for anemia reduction, and its use could be scaled up. To prepare for possible expanded use and adoption of IV iron for treatment of postpartum anemia, data related to the implementation of the intervention in LMICs is needed. Two trials with accompanying implementation research (IR) on FCM IV iron in pregnant women with moderate or severe iron deficiency anemia were recently completed in Nigeria and Malawi and one hybrid effectiveness-implementation trial in postpartum women with moderate or severe anemia is in progress in Nigeria. ^13– 18^ To our knowledge, there are no implementation studies on FCM IV iron postpartum in South Asia. To fill this gap, we will conduct an IR study alongside the PRIORITY trial in two of the sites – India and Pakistan – where anemia prevalence in 2019 was 53% and 41%, respectively. ^19^

The aims of the PRIORITY IR study are to: (1) gather information on IV iron intervention characteristics, the settings in which it is implemented, characteristics of the individuals involved in implementation, and the implementation process, and (2) assess the feasibility, acceptability, fidelity, and cost of implementation. The findings will be useful for potential future scale up of the intervention in India and Pakistan and for informing global guidelines on the use of IV iron in postpartum women in LMICs, should the main PRIORITY trial and other on-going trials demonstrate intervention effectiveness.

## Protocol

### Study design

**Figure 1.**
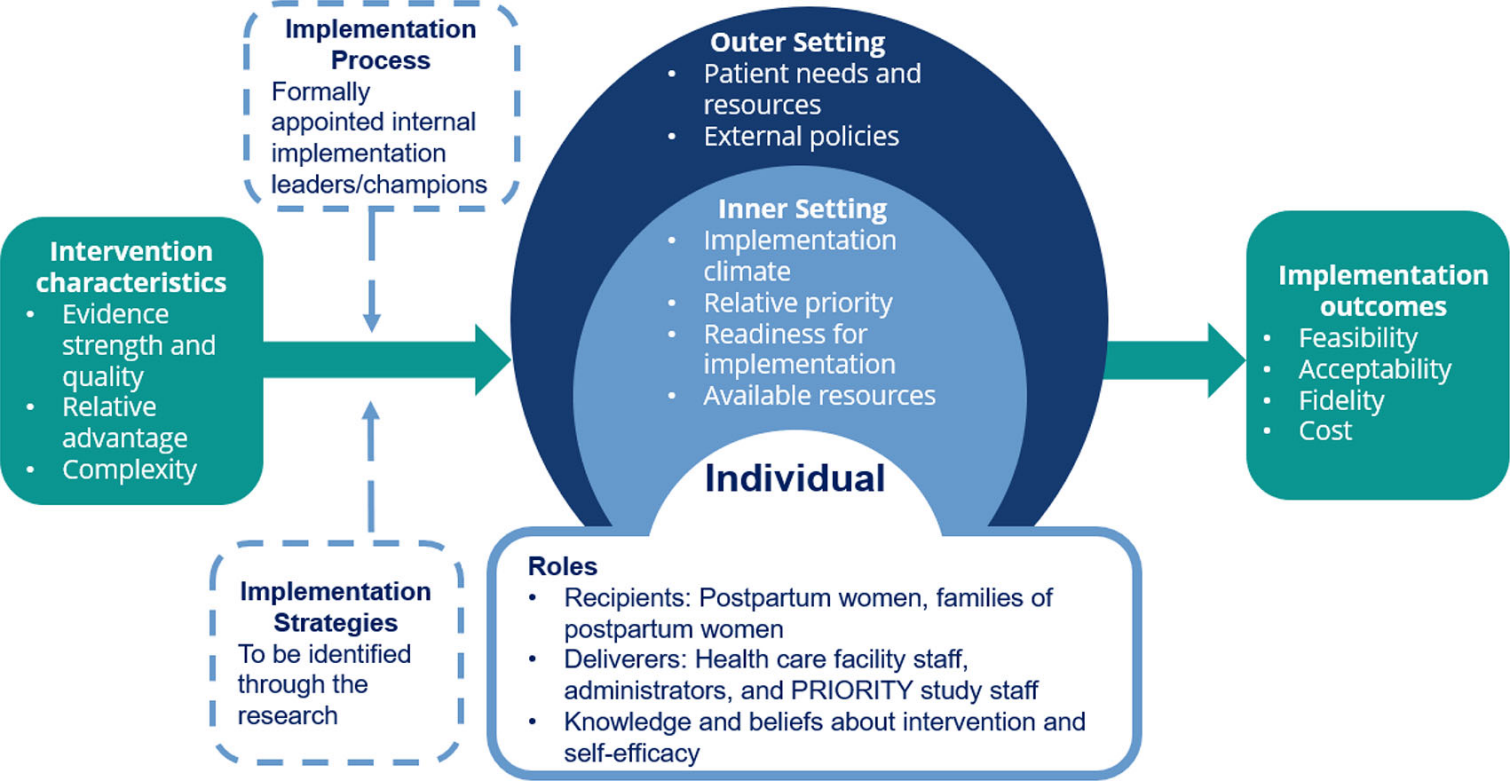
CFIR domains and constructs and Proctor’s implementation outcomes used in the PRIORITY IR study. Image based on constructs in Damschroder et al. 2009 ^20^ and Proctor et al. 2011 ^22^ and adapted from The Center for Implementation 2022, https://thecenterforimplementation.com/toolbox/cfir.

This mixed methods study will be guided by the Consolidated Framework for Implementation Research (CFIR) and by Proctor’s implementation outcomes. ^20– 22^ The model in Figure 1 represents a synthesis of the CFIR domains and constructs as well as the implementation outcomes that will be used in the PRIORITY IR study. The CFIR framework will be used to assess the intervention characteristics, outer setting, inner setting, characteristics of individuals, and implementation process related to the IV iron intervention. Proctor’s framework will be used to assess feasibility, acceptability, fidelity, and cost and to document implementation strategies that may inform adaptations. CFIR and Proctor’s framework will be complementary and provide a structure for guiding the types of questions and target groups for IR data collection.

### Study setting

The study will be conducted in Belagavi, India and Karachi, Pakistan study sites and will include all health facilities at those sites that are part of the PRIORITY trial. In Belagavi, the data will be collected by the Jawaharlal Nehru Medical College team at three sub-district hospitals in Hukkeri, Bailhongal, and Gokak in Karnataka State. In Karachi, the data will be collected by the Aga Khan University (AKU) at the Jinnah Postgraduate Medical Centre.

### Participants and eligibility criteria

In both sites, study participants will include women randomly assigned to the IV iron intervention arm of the PRIORITY trial, family members (i.e., husbands and mothers or mothers-in-law of women assigned to IV iron intervention arm), health workers who administer IV iron infusions as part of the PRIORITY trial, and hospital administrators at the study sites.

Women in the IV iron arm will be interviewed to understand their decision-making related to participating in the trial, their experience of receiving IV iron, and their perceptions about the intervention. Family members will be included to understand their perceptions of the women’s experience with IV iron and interactions with health staff delivering the intervention. Health workers and hospital administrators will be included to understand their perceptions of the intervention characteristics and how the inner and outer settings influences implementation. Health workers will also be asked about their implementation strategies for delivering the intervention and their perceptions and experiences related to providing IV iron. Hospital administrators will be asked about factors that would influence the hospital’s decision to provide IV iron, resources needed, and facilitators and barriers to implementation from their perspective.

 Site-specific participants will include women who refuse to participate in the PRIORITY trial in Pakistan and women in the oral iron arm of the PRIORITY trial in India. Our initial plan was to include women who refuse to participate in the PRIORITY trial at both sites. However, the institutional review board (IRB) in India did not consider it ethical to conduct a short survey (with consent) with women who refused to be in the main trial. The IRB in India also requested the addition of women in the oral iron arm to the six-month postpartum exit interview that was planned for women in the IV iron arm as part of the IR study. To comply with these requests from the IRB, the PRIORITY IR study will include two site-specific groups. In Pakistan, women who refuse to participate in the PRIORITY trial will be enrolled as IR study participants to understand why they chose not to participate. In India, women enrolled in the oral iron arm of the PRIORITY trial will be included as IR study participants and will participate in a short interview along with women in the IV iron arm to gather information about participants’ experiences with the interventions at the time of exit from the PRIORITY trial.

Eligibility criteria are outlined below.

**Figure 2.**
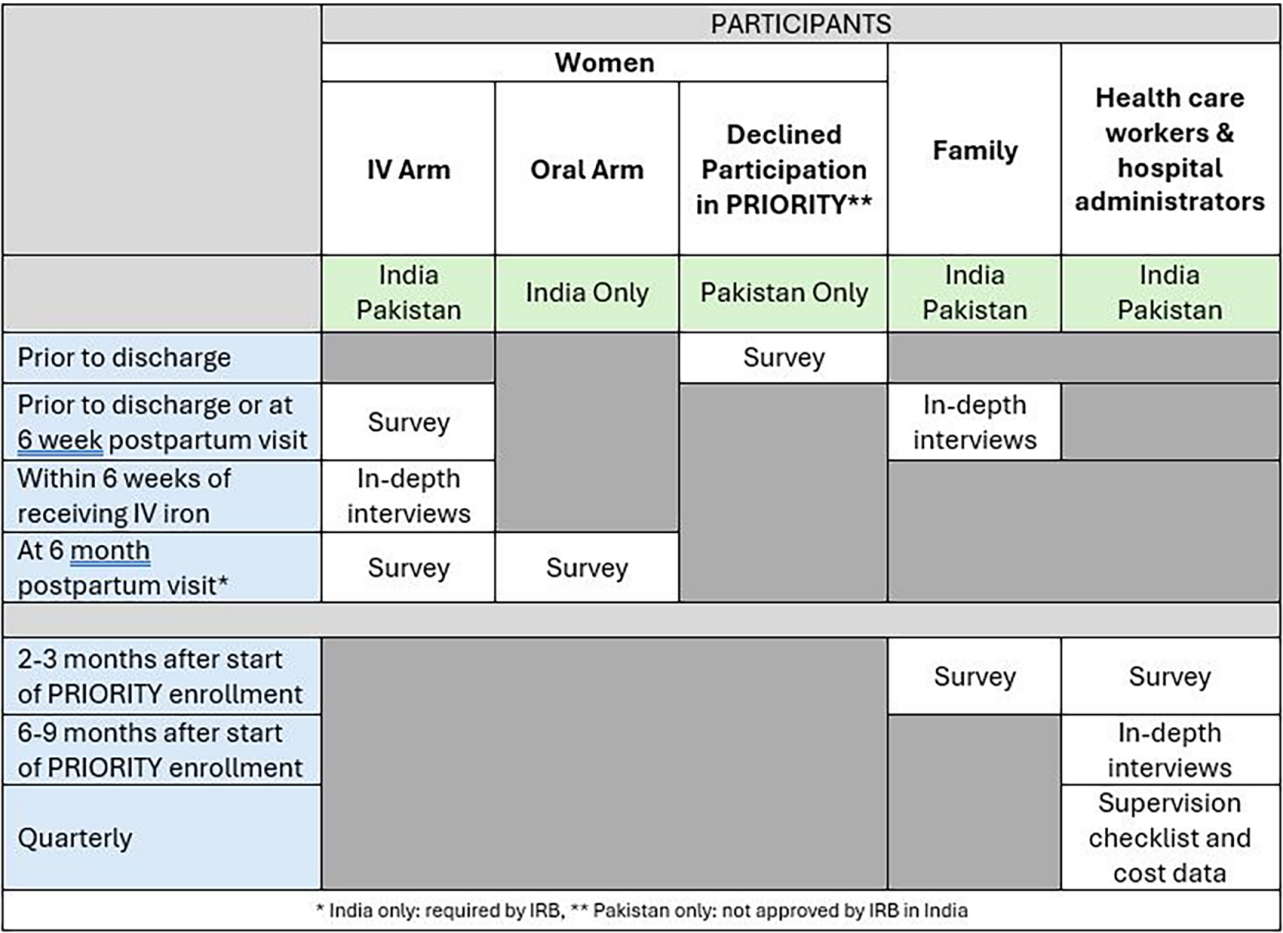
PRIORITY IR participants, data collection methods, and timing of data collection.

Women:

• Recently delivered
• Aged 18-45 years in India or 15-45 years in Pakistan
• Confirmed moderate anemia
• No contraindication to iron supplementation
• Have not received and are not scheduled to receive a blood transfusion
• Consent to participate in PRIORITY and/or IR study (see details in Figure 2)

Family members (husbands, mothers, or mothers-in-law) of women enrolled in the IV iron arm of the PRIORITY trial:

• Age ≥ 18 years
• Consent to participate in the IR study

Family members who are involved in decision-making about the woman’s participation will be prioritized for inclusion.

Health workers who administer IV infusions as part of the PRIORITY trial and hospital administrators (such as the CEO, department head, unit/section heads, or hospital manager) responsible for implementation decisions and involved in the PRIORITY trial:

• Age ≥ age 18 years
• Consent to participate in the IR study

### Study procedures

Categories of study participants, associated data collection methods, and timing of data collection are summarized in Figure 2. Sample sizes by data collection method for different types of participants are shown in Table 1.

**Table 1.** Sample sizes by data collection method and participant type.

Data collection method	Participant type	Sample size
Surveys	Women enrolled in IV iron arm of PRIORITY trial	Up to 300 per site
	Health workers	Up to 12 per site
	Hospital administrators	Up to 6 per site
	* Pakistan only:* Women who refuse to participate in PRIORITY trial	Up to 50
	* India only:* Women in oral iron arm of PRIORITY trial	Up to 300
In-depth interviews	Women enrolled in IV iron arm of PRIORITY trial	Up to 15 per site
	Family members (husbands, mothers, mothers-in-law) or women enrolled in IV iron arm of trial	Up to 15 per site
	Health workers	Up to 12 per site
	Hospital administrators	Up to 6 per site
Supervision checklist	Health workers	2 per health worker per quarter

*Screening and enrollment*

All women enrolled in the PRIORITY trial and randomly assigned to the IV iron intervention group will be invited to participate in the IR study and will be enrolled by trained research staff. A sub-sample of women in the IV iron arm of the PRIORITY trial will be selected to participate in in-depth interviews (IDIs). A single consent form will be used for all IR study activities for women enrolled in the PRIORITY trial. Women enrolled in the PRIORITY trial will be asked to connect the study team to their family members (i.e., husbands, mothers, or mothers-in-law) if they feel comfortable doing so.

A list of hospital workers and hospital administrators involved in providing or overseeing the administration of IV iron in the PRIORITY trial will be compiled by the sites. Identified hospital workers and hospital administrators will be asked to enroll in the IR study by trained research staff members who are not clinicians or administrators. PRIORITY trial study staff will not be included in the study population for the PRIORITY IR study.

In the Pakistan site only, women who refuse to participate in the PRIORITY trial will be asked to complete an interview prior to discharge from the hospital. In the India site only, all women enrolled in both arms of the PRIORITY trial will participate in a short exit survey at 6 months postpartum during their last PRIORITY trial study visit.

*Data collection methods and data management*

Data will be collected by trained study staff through surveys with PRIORITY trial women; women who refuse to participate in PRIORITY (Pakistan site only); hospital staff and administrators; IDIs with PRIORITY trial women and their family members and with hospital staff and administrators; supervision of hospital staff performing IV iron provision tasks; and tabulation of costs. Data collection tools will be translated into Kannada and Urdu in India and Pakistan, respectively. Surveys and qualitative interviews will be conducted in these languages.

Training for study staff will include discussion of study procedures, review of the data collection forms, and a pilot test of data collection. During the training, data collection forms will be revised to improve question flow, remove redundancies, and simplify question wording to facilitate translation and interpretation of questions by participants. All study staff will complete an approved ethics training prior to participating in the training for the IR study.

Surveys will be collected electronically using the Research Electronic Data Capture (REDCap) mobile application on Android tablets. Data entered offline into REDCap mobile app will be uploaded to the Research Triangle Institute (RTI) REDCap server daily or as soon as there is a reliable internet connection. For PRIORITY trial participants who participate in the IR, the same study ID used for the trial will be used for the IR study. All other IR study participants will be assigned new unique study IDs. The RTI data coordinating center will provide support on the data management system and will maintain the central database for the study.

Qualitative data from IDIs will be collected by trained moderators and notetakers. IDIs will be electronically recorded using a digital voice recorder. The recordings will be uploaded to a secure server at each site and deleted from the digital recorders on the same day as the data is collected. Notetakers during IDIs will take handwritten notes and type them on the same day as data collection, which will help the transcriptionist and will aid the early analysis and development of a list of themes and codes.

Demographic data for the PRIORITY trial participants involved in the IR study will be obtained from data collected as part of the trial. For other IR study participants, basic demographic data (such as age and sex) will be collected from participants as part of the IR study.

Cost data will be collected for all PRIORITY trial sites. The IR study team will review cost data for only the Belagavi and Pakistan sites to understand the financial burden of IV iron administration on women and the healthcare system. Cost data will include the total cost for the IV iron administration delivery and the average IV iron administration delivery costs per woman. Additionally, data from Belagavi and Pakistan sites’ supervisor checklists used in the main PRIORITY trial to oversee administration of IV iron will be used as part of the PRIORITY IR study to understand fidelity of administration of IV iron.

*Ethical considerations*

A consent form will be read to each participant by trained research staff to inform them of the study’s purpose and its risks and benefits. The participant will have an opportunity to ask questions about the IR study and have their questions answered. If the participant does not want to participate in the study, they can refuse without any penalty. Participants can also agree to participate and then withdraw at any time. Participants will be asked to consent to have their de-identified data included as part of U.S. National Institutes of Health (NIH) public-use databases in the public forum ( clinicaltrials.gov) in compliance with the NIH Public Access Policy.

The study protocol, informed consent forms and any modifications, will be reviewed and approved by the IRBs of all participating sites as follows: Thomas Jefferson University IRB (2023/03/25; iRISID-2023-1605); Institutional Ethics Committee, KLE Academy of Higher Education and Research, Belagavi, India (2023/05/24; KAHER/EC/2023-24/d-24052307); AKU (2023/06/13; 2023-8510-25288); and RTI (2023/07/28; 12940-9a). Participants will be asked to provide written informed consent. They will sign two copies of the consent form and will be given one copy to keep.

All electronic data forms, reports, and other records that leave the site will be identified only by a coded ID number to maintain participant confidentiality. IDI transcripts will be prepared without including participant identifiers. IDI participants will be referred to in the transcripts only by their study ID numbers. All records entered into REDCap web and mobile app will be encrypted and password protected at all times. Only authorized staff will have access to study data, including private health information, for study identification and follow-up purposes.

Women and family members who participate in the IDIs will be compensated for their participation with a small payment that includes loss of daily wages, travel to site, and lunch, as per each site’s incentive guidelines. Hospital workers and hospital administrators involved as participants in the IR study will continue to receive their usual salaries from their employers without receiving additional incentives from the study.

### Data analysis

In this mixed methods study, we will use a convergent parallel design, ^23^ where quantitative and qualitative data will be independently analyzed then compared to identify convergence and divergence of findings, providing the team with a more holistic understanding of barriers and facilitators to postpartum IV iron implementation.

Quantitative data from multiple data sources will be analyzed quarterly by an RTI statistician using SAS statistical software (version 9.4, SAS Institute, Cary, NC, https://www.sas.com/en_us/software/stat.html). The RTI study team will report frequencies for categorical data and calculate percentages (e.g., percentage of health workers who follow the procedures for implementing the intervention). The India and Pakistan study teams will analyze the qualitative data in NVivo software (version 14.23.4, Lumivero, Denver, CO, https://lumivero.com/products/nvivo/) using directed content analysis methods ^24^ with guidance and support from Thomas Jefferson University. Codebooks will be developed in two ways: deductive codes using the CFIR and Proctor frameworks and inductive codes through line-by-line reading of a subsample of interview transcripts. Each code will be given an explicit definition to ensure coding accuracy and improve intercoder reliability. Coding will be supervised by a qualitative research scientist. Any coding discrepancies will be resolved through consensus. The coded data will be used to complete data matrices in Excel with themes in the columns and participant ID numbers in the rows. ^25^ Illustrative quotes will be inserted into the cells of the matrix to facilitate analysis. Qualitative and quantitative results will be used to describe implementation outcomes (e.g., feasibility, acceptability, fidelity of interventions and implementation cost and strategies) and experiences of anemic women, their family members, health workers, and other hospital staff.

The qualitative analysis process will establish trustworthiness through the research and analysis process. ^26^ We will increase credibility of the findings by triangulating results from the qualitative and quantitative data and through investigator triangulation. The inclusion of two sites in our study increases transferability because it allows for comparing and contrasting findings in two settings. We will ensure dependability by creating a traceable process of code development, application, and subsequent analysis. Confirmability will be assured by having a systematic approach to interpreting the findings, presenting different perspectives on a theme or construct if they are present in the data, and through triangulation.

### Stakeholder engagement

We plan to engage with stakeholders at the local, national, and international levels to disseminate findings and provide information from this study that can shape policies in coordination with findings from the main trial. Engagement will take place through individual discussions with policymakers and through technical consultations related to maternal and newborn health and anemia in the two countries and with global organizations and funders, such as the World Health Organization and Gates Foundation.

## Conclusions/Discussion

The prevalence of anemia remains high among women of reproductive age, especially in LMICs, and there has been insufficient progress toward global anemia reduction goals. ^3^ Reducing iron deficiency anemia postpartum is an important part of anemia reduction strategies because it compounds anemia during pregnancy with subsequent blood loss during delivery, which may result in negative health consequences for mothers and their infants. ^1, 2^ Recent reviews and meta-analyses have shown the benefits of employing IV iron to treat anemia because of the rapid increase in hemoglobin and the safety of the approved FCM formulations. ^9, 10^ The single dose administration and lower side effects of IV iron also contribute to better adherence compared with oral iron.

The PRIORITY trial will collect data on treatment response and health outcomes of a single dose of FCM IV iron compared with oral iron in sites across three continents. The findings will likely influence global policy, which could lead to changes in the standard of care for postpartum anemia in LMICs. The PRIORITY IR study will contribute important information about the feasibility, acceptability, fidelity, and costs of implementation and will document implementation processes and strategies. These findings together with results from IR in IV FCM trials in Nigeria and Malawi ^16, 18^ can be used to provide accompanying guidance on best practices for implementation across LMIC contexts where anemia among women of reproductive age is highest.

## Author contributions

All authors contributed to the preparation of the protocol for institutional review board submission. VLF drafted the manuscript. All authors reviewed the manuscript draft and final versions.

## Data Availability

No data are associated with this preprint.

## Software availability

The quantitative analysis will be performed using SAS software but could be done using an equivalent function in R ( https://www.r-project.org/). The qualitative analysis will be performed using NVivo software but could be done using equivalent coding procedures in QDA Miner Lite ( https://provalisresearch.com/products/qualitative-data-analysis-software/freeware/).
